# A Core Outcome Set and minimum reporting set for intervention studies in growth restriction in the NEwbOrN: the COSNEON study

**DOI:** 10.1038/s41390-020-01119-5

**Published:** 2020-09-14

**Authors:** Stefanie E. Damhuis, Frank H. Bloomfield, Asma Khalil, Mandy Daly, Wessel Ganzevoort, Sanne J. Gordijn

**Affiliations:** 1 Department of Obstetrics and Gynaecology, University Medical Center of Groningen, University of Groningen, Groningen, The Netherlands; 2 Liggins Institute, University of Auckland, Auckland, New Zealand; 3 Fetal Medicine Unit, St. George’s University and St. George’s University Hospitals, NHS Foundation Trust, London, UK; 4 Irish Neonatal Health Alliance, Bray, Ireland; 5 Department of Obstetrics and Gynaecology, University Medical Centers Amsterdam, University of Amsterdam, Amsterdam, The Netherlands

## Abstract

**Background:**

Different interventions and treatments are available for growth-restricted newborns to improve neonatal and long-term outcomes. Lack of outcome standardization across trials of feeding interventions limits pooled analysis of intervention effects. This study aimed to develop a core outcome set (COS) and minimum reporting set (MRS) for this research field.

**Methods:**

A scoping search identified relevant outcomes and baseline characteristics. These outcomes were presented to two stakeholder groups (lay experience and professional experts) in three rounds of online Delphi surveys. The professional experts were involved in the development of the MRS. All items were rated for their importance on a 5-point Likert scale and re-rated in subsequent rounds after presentation of the results at the group level. During a face-to-face consensus meeting the final COS and MRS were determined.

**Results:**

Forty-seven of 53 experts (89%) who completed the first round completed all three survey rounds. After the consensus meeting, consensus was reached on 19 outcomes and 17 baseline characteristics.

**Conclusions:**

A COS and MRS for feeding interventions in the newborn after growth restriction were developed. Use of these sets will promote uniform reporting of study characteristics and improve data synthesis and meta-analysis of multiple studies.

**Impact:**

- Both a COS and MRS for growth restriction in the newborn were developed.
- This study provides the first international combined health-care professional and patient consensus on outcomes and baseline characteristics for intervention and treatment studies in growth-restricted newborns.
- The use of COS and MRS results in the development of more uniform study protocols, thereby facilitating data synthesis/meta-analysis of multiple studies aiming to optimize treatment and interventions in growth restriction in the newborn.

## Introduction

Growth restriction in the newborn (GRN) is the newborn equivalent of fetal growth restriction (FGR).^1^ Suboptimal growth is associated with poor outcomes regardless of the timing of diagnosis.^2–4^ After the birth of a growth-restricted newborn, different interventions and treatments, predominantly feeding strategies, have been described and each of these interventions has its own/specific benefits and risks.^5–8^ Positive immediate effects of successful feeding, such as accelerated (“catch-up”) growth, can, however, have a potential negative trade-off for metabolic health, including a higher risk of obesity and cardiovascular disease.^9–12^ Given the increased risk for neonatal morbidity and mortality in GRN and the increased risk for poor neurodevelopmental outcomes later in life, evidence is needed on the most effective and safest interventions and treatments for these growth-restricted newborns.

Comparing results from interventional and treatment studies concerning GRN is hampered due to the use of different baseline characteristics and, equally important, differences in reported study outcomes.^13^ In order to be able to compare study results and to allow pooling of data, more and more attention is paid to the development and use of core outcome sets (COSs). The value of COSs can be greatly enhanced by adding a set of essential baseline characteristics to report, often referred to a minimum reporting set (MRS), in order to align knowledge about details of the study populations and circumstances.^14^ COSs are agreed, clearly defined, minimum sets of outcomes that can be measured in a standardized manner and reported consistently.^15^ Standardization of outcomes for clinical trials ought to prevent the problems of inappropriate and non-uniform outcome selection and reporting bias.^15,16^ Both an MRS and a COS have already been developed for FGR.^14,17^

The aim of this study was to develop both a COS and an MRS for GRN using the Delphi methodology engaging key stakeholders.

## Methods

The international steering group consisted of healthcare professionals, researchers, and patient representatives. A protocol with explicitly defined objectives, consensus methods, participant recruitment, and statistical methods was developed and prospectively registered at the Core Outcome Measures in Effectiveness Trials (COMET) initiative (registration number: 1001) and published.^18,19^ The ethical board of the University Medical Center of Groningen provided a waiver of an ethical approval procedure (reference number: METc 2018.624).

### Scope

Both the COS and MRS apply to postnatal non-invasive therapeutic interventions and treatments for growth-restricted newborns, either diagnosed antenatally (FGR) or at birth (GRN), defined according to international consensus definitions.^1,20^ Both sets concern the specific items of growth restriction, regardless of gestational age. For preterm birth a COS has already been developed.^21^

### Literature search

A scoping literature review was performed for published literature on intervention and treatment studies of GRN in PubMed from 2008 to June 2018 with search terms diet therapy (nutritional management, feeding, diet, diet therapy) and fetal growth retardation (small for gestational age, SGA, intrauterine growth restriction, very low birth weight, fetal growth restriction, FGR, growth-restricted fetuses, fetal growth retardation). Titles and abstracts of clinical trials were screened and baseline characteristics and reported outcomes were extracted. Articles were included if they concerned term and/or preterm GRN and nutritional management intervention. Exclusion criteria were articles concerning preterm but not specifically growth-restricted newborns, treatment by hormones or medication, parentally administered treatment, or when the article was not available in English. Any discrepancies were resolved by discussion within the steering group.

### Stakeholders

Two stakeholder groups were involved in this study: a lay expert group consisting of patient representatives and parents of growth-restricted newborns, and a stakeholder group consisting of professional experts, including neonatologists, (general) pediatricians, obstetricians, and researchers with a special interest in GRN.^1^ Lay experts were approached through patient forums and through posters and flyers that were displayed in the public areas of participating hospitals (obstetric and pediatric wards as well as the Neonatal Intensive Care Units), general practitioner practices, and midwifery clinics located in the Netherlands and in London. The poster and flyer material contained both a link and QR code to subscribe to the procedure and a link to a short video with explanations in plain language of the background and aim of the project by a neonatologist (F.H.B.). All communication was in English.

### Delphi study

An electronic three-round survey was performed, in which the Delphi consensus methodology was applied; details of the planned procedure have been published.^19^ The Delphi procedure aims for convergence of opinions resulting in consensus of participants by multiple rounds, wherein statements are weighed, summarized, and fed back at the stakeholder level (individual answers are anonymous).^22^ The survey was subdivided into a procedure to come to a COS and one to come to an MRS. In the latter, only professional experts were involved. Both the COS and MRS were structured into several domains. A description in plain language of each outcome was provided in the online surveys.

In the first round, all participants were asked to rate the importance of each outcome on a 5-point Likert scale anchored between 1 (very unimportant) and 5 (very important). For the development of the MRS, only the professional experts were asked to rate the importance of each baseline characteristic. The predefined cut-off for inclusion was a median Likert score of 5. All participants could suggest additional outcomes and professional experts were also asked to recommend additional missing baseline characteristics. Outcomes and baseline characteristics suggested at least twice by individual participants were reviewed by the steering group for presentation in the next round.

In the second round, the medians of all outcomes were presented at the stakeholder group level. Participants were asked to reflect their opinion of the importance of each outcome in the light of these results and possible differences between the stakeholder groups and to score each outcome again. Professional experts were also asked to re-rate each baseline characteristic with the medians of all baseline characteristics, presented at the group level, in mind.

In the third round, outcomes were presented to confirm inclusion when >70% of both panels scored an outcome Likert score of 5 and ≤15% of both panels scored an outcome Likert score of 1, or when >90% of a single panel scored an outcome Likert score of 5. Baseline characteristics were presented to confirm inclusion when >70% of the experts scored a characteristic Likert score of 5. Outcomes and baseline characteristics that did not meet the predefined criteria as described above were presented for verification of exclusion.

The results of the Delphi survey were discussed in a face-to-face consensus meeting organized as a satellite to a large international meeting in order to achieve an international representation of participants. All participants received a summary of the results of round 3 prior to the meeting. The meeting was chaired by a non-voting member of the steering committee with expertise in the development of a COS (W.G.). Outcomes identified in round 3 of the Delphi as having reached consensus for inclusion were presented first and participants were asked if there were any fundamental reasons why these should not be included in the COS. All outcomes for which no consensus was reached were discussed and voted on, one item at a time. The chair ensured all participants had an equal opportunity to contribute before voting commenced. The consensus was defined with a pre-set level of agreement of five out of six participants (>83% agreement) to finalize the COS and three out of three participants (100%) in the procedure to finalize the MRS. Consensus rules were strictly adhered to and, following discussion, voting was done anonymously with blinded jars that collected masked papers with “yes” and “no”.

The final COS was presented to all participants who completed round 3 and they were asked to indicate whether they agreed upon the eventual set of outcomes. The same principle was applied for the MRS in which the professional experts could agree or disagree upon the set of characteristics. In case of disagreement on a specific outcome or baseline characteristic, the participants were asked to provide a reason.

### Data collection

Data were collected using online questionnaires. The responses were captured in the online REDCap tool, version 7.3.2. Every participant received a unique token-secured link to participate in the online survey. Participants received at least two reminder emails, and non-responders were excluded from subsequent survey rounds.

## Results

**Fig. 1:**
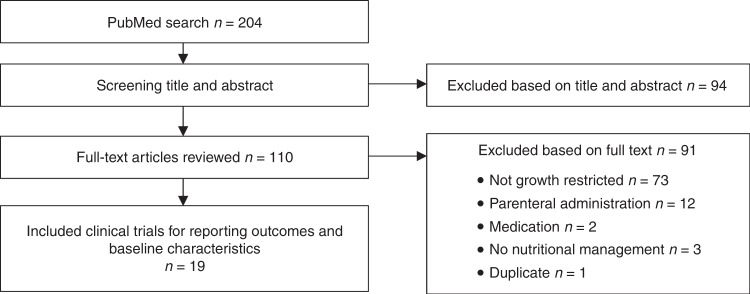
Flow chart of study selection. Flow chart of study selection for identifying outcomes and baseline characteristics.

The PubMed scoping search yielded 19 clinical intervention trials. One hundred and sixty-four outcomes were identified and 121 baseline characteristics (Fig. 1). After checking with the steering group for relevance to the study population, missing items, doubling, and overlap, 77 outcomes and 48 baseline characteristics were grouped into domains and presented to the stakeholders in the online survey rounds.

One hundred and fifty-one invitations were sent in the first round, of which 111 were for participants who had registered their interest for the procedure and 40 professional experts were approached directly and invited to participate. Fifty-three participants completed the first round, of whom 34 were professional experts and 19 were parents of GRN. A total of 47 participants (88.7%) completed all three rounds. Demographic characteristics of these participants are shown in Table 1a, b.

**Table 1:** (a) Characteristics of lay experts (patient representatives and parents of a growth-restricted newborn) who completed all three online rounds. (b) Characteristics of professional experts who completed all three online rounds.

	Number	Percent
(a) Parent characteristics		
Sex		
Male	2	14.3
Female	12	85.7
Region of domicile		
Europe	14	100.0
North America	0	0
South America	0	0
Asia/Australia	0	0
Africa	0	0
Time since GRN was born		
<5 years	10	71.4
5–10 years	2	14.3
10–15 years	0	0
>15 years	2	14.3
(b) Professional experts’ characteristics		
Occupation		
Neonatologist	25	75.8
(General) pediatrician	3	9.1
Obstetrician/gynecologist	3	9.1
Researcher	2	6.1
Sex		
Male	16	48.5
Female	17	51.5
Country of practice		
Europe	22	66.7
North America	5	15.2
South America	0	0
Asia/Australia	6	18.2
Africa	0	0
Professional position		
Professor	12	36.4
Associate professor	7	21.2
Assistant professor	0	0
Consultant/medical specialist	13	39.4
Fellow	0	0
Registrar/trainee	1	3.0
Non-clinical investigator/researcher	0	0
Midwife	0	0
Other	0	0
Years of practice in current function		
0–4 years	1	3.0
5–9 years	10	30.3
10–20 years	9	27.3
>20 years	13	39.4
Tertiary referral center for FGR/GRN		
Yes	28	84.8
No	5	15.2
Annual births at expert’s center		
<1000	0	0
1000–2500	9	27.3
2500–5000	9	27.3
>5000	12	36.4
Unknown	3	9.1
GRN treated by individual expert on annual base		
<5	0	0
5–15	1	3.0
15–25	5	15.2
>25	20	60.6
None	2	6.1

**Fig. 2:**
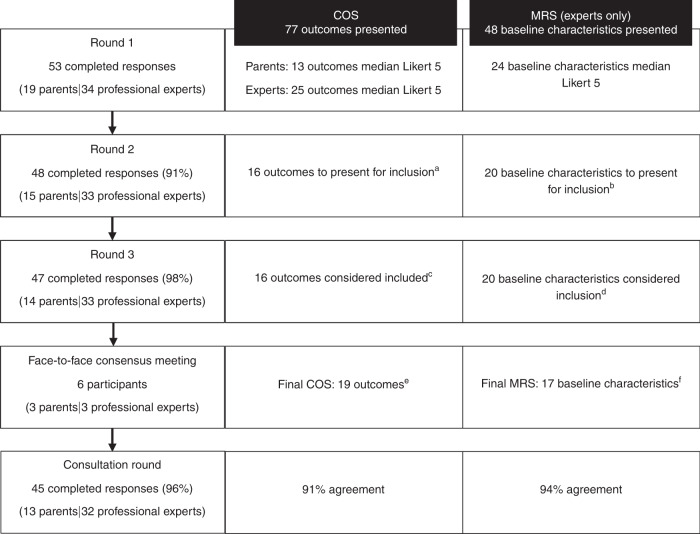
Summary of study methods and results. ^a^Both stakeholder groups >70% with a Likert score of 5 and <15% with a Likert score of 1 OR one stakeholder group >90% with a Likert score of 5. ^b^Greater than 70% with a Likert score of 5 and <15% with a Likert score of 1 (professional experts only). ^c^Agreement on inclusion >70 in both stakeholder groups or >90% in one stakeholder group. ^d^Agreement on inclusion >70% (professional experts only). ^e^Consensus criteria: five out of six agreement (>83%). ^f^Consensus criteria: three out of three agreement (100%).

In the first round, 13 outcomes were scored a median Likert score of 5 (very important) by the parents and 25 outcomes were scored a median Likert score of 5 by the professional experts. Twenty-four baseline characteristics scored a median Likert score of 5. No new outcomes or baseline characteristics were added after round 1 as none of the suggestions was mentioned by two or more individual participants. In the two subsequent electronic rounds, all outcomes and baseline characteristics were brought back for consensus on inclusion. Ultimately, a total of 16 outcomes and 20 baseline characteristics met the criteria for inclusion after round 3 (Fig. 2).

The face-to-face consensus meeting was held as a satellite meeting to the joint European Neonatal Societies (jENS) conference in Maastricht, the Netherlands on 21 September 2019. Three participants from each stakeholder group and from four countries participated. Consensus decisions made during this meeting are shown in Supplementary Table S1. The final COS includes 19 outcomes across eight domains (Table 2) and the final MRS includes 17 baseline characteristics across five domains (Table 3). Outcomes and baseline characteristics not included in the eventual sets are shown in Supplementary Tables S2 and S3.

**Table 2:** Final COS to be included in all studies on GRN.

Domain	Outcome
Gastrointestinal	Necrotizing enterocolitis
	Need for gastrointestinal surgery
Respiratory	Bronchopulmonary dysplasia
Neurological	Intraventricular hemorrhage
	Periventricular leukomalacia
Infection	Sepsis
Feeding	Breastfeeding
Hospital and mortality	Duration of stay in a newborn nursery (days)
	Hospital stay (days)
	Need for neonatal intensive care
	Neonatal mortality
Growth/weight	Head circumference
	Length
	Weight gain
Long-term follow-up	Cerebral palsy
	Cognitive impairment
	Hearing impairment
	Motor impairment
	Visual impairment

**Table 3:** Final MRS to be reported in all studies on GRN.

Domain	Baseline characteristic
Maternal baseline characteristics	Age (years)
	BMI (pre-pregnancy or first trimester)
	Highest level of education
Obstetric information	Substance abuse during pregnancy^a^
Complications antenatal	Congenital infection likely to affect fetal growth
	Hypertensive disorders during pregnancy^b^
General information postpartum	Antenatally detected fetal growth restriction
	Birth weight (g)
	Gestational age at the time of delivery
	Sex of the newborn
	(Part of) multiple pregnancy
	Mode of birth
	Chromosomal abnormalities
	Congenital anomalies likely to affect fetal growth
	Head circumference at birth (cm)
	Length at birth (cm)
Fetal baseline characteristic	Hypoxic–ischemic encephalopathy

^a^Smoking, alcohol, and drugs.

^b^Including gestational hypertension, preeclampsia, and Hemolysis, Elevated Liver enzymes, and Low Platelets syndrome (HELLP).

The consultation round was completed by 45 (95.7%) of the participants completing round 3, of whom 41 (91.1%) agreed with the final COS and 30 (93.8%) of the 32 expert professionals agreed with the final MRS.

## Discussion

A COS and an MRS for intervention and treatment studies in GRN were developed using the Delphi consensus methodology. They consist of 19 outcomes and 17 baseline characteristics, respectively. Both outcomes and baseline characteristics can be used in the development of future study protocols, conduction of reviews, and guidelines on GRN. It is important to note that COS and MRS represent the minimum set of outcomes and baseline characteristics that should be reported in all trials on that specific research topic. The list is not exhaustive and additional outcomes and baseline characteristics should be freely reported if deemed relevant.^15^

The COS captures meaningful outcomes of GRN, including morbidity of several organ tracts, growth, mortality, and long-term follow-up. Some of the included outcomes merit further explanation. First, periventricular leukomalacia (PVL) is hard to measure and needs extra investigations in the newborn. Inclusion of PVL should not oblige future studies to perform standard investigations to assess PVL, but should be measured and reported when undertaken as part of the study and, when not assessed, this should be stated. Second, the outcomes “hospital stay” and “NICU stay” are subject to significant practice variation and to factors that are not strictly related to the disease burden of the newborn. For example, cesarean section prolongs maternal hospital stay (and thus neonatal hospital stay regardless of the neonatal condition) compared to vaginal birth, and different newborn services will have different policies and protocols about when to admit babies to the nursery. Therefore, researchers are encouraged to report the indication(s) for neonatal “hospital stay” and “NICU stay” recognizing that they are strongly correlated, but are considered to cover a slightly different representation of the disease burden. After lengthy discussions during the consensus meeting, it was decided to include both outcomes “hospital stay” and “NICU stay” as these are easy to measure and provide insight into the circumstances of the study, the intervention results, and potential cost savings.

One of the included outcomes is breastfeeding, indicating that for both healthcare experts and patients whether or not the infant is (fully) breastfed during and/or after interventions and treatments is considered very important. Of note, the baseline characteristic “intends to breastfeed” was voted out from the MRS. However, it was discussed that the MRS represents a *minimum* of what should be reported at baseline in all studies on GRN. Another baseline characteristic not included was the “use of assisted reproductive technology” (ART) because these data may be challenging to report reliably, although the literature suggests that certain variables in ART might influence birth weight.^23,24^ Reporting this baseline might be considered if deemed relevant.

### Strengths and limitations

For the development of the COS, the guidelines outlined by the COMET initiative were used.^18^

Recruitment of parents and patient representatives occurred via posters, patient forums, folders, and newsletters of patient representatives. This form of recruitment led to an almost 100% representation of Dutch participants in the patient stakeholder group. Despite the potential risk of selection bias due to possible higher educational level, the fact that lay experts were involved as stakeholder group was considered as an important strength of the study. Lay expert involvement is a crucial contribution in developing a COS as outcomes that are most relevant to patients or carers, the ones who actually feel the burden and benefits of the interventions and treatments, should be included.^18^ The scores of both stakeholder groups were weighed equally even though the number of participating lay experts was smaller than the number of participating professional experts.

Another limitation is the limited number of participants in the face-to-face meeting. This was due to the requirement of equal representation of both stakeholder groups and the inability to achieve attendance of more than three lay experts. We think that this limitation was significantly attenuated by the high agreement in the online panel of participants in the consultation round.

A scoping review instead of a systematic review was performed with the rationale that an outcome that is hardly ever reported is unlikely to be part of a COS. This rationale was strengthened by the fact that although participants were asked to suggest additional outcomes and baseline characteristics not listed in the initial list of outcomes and baseline characteristics, no consistent additional suggestions were made. Pre-set inclusion rules allowed that when >90% of one of the stakeholder groups scored an outcome Likert score of 5, it would be taken forward as an inclusion to the next round, regardless of the other stakeholder group score.

Due to financial constraints, only a face-to-face consensus meeting was held (and no additional electronic meeting as planned in the protocol).

### Final COS/MRS

During the consensus meeting, three outcomes were added to the final COS and one outcome “hypoxic–ischemic encephalopathy (HIE)” was considered to be a baseline characteristic as it happens prior to, during, or shortly after birth, and is thus either present or absent in the growth-restricted newborn. HIE was previously included as an outcome in the developed COS for the antenatally detected growth restriction.^17^ Two baseline characteristics (premature rupture of membranes and placental abruption) were excluded from the final COS as both were not considered to provide relevant information about the effect of treatments in GRN.

Both the final COS and MRS corresponds largely with the previously developed COS and MRS for FGR.^17^ Some baseline characteristics incorporated in the current MRS were included as outcomes in the COS of FGR, which is logical as the change of outcome to baseline characteristic is at birth. For example, birth weight is an outcome in the antenatal diagnosis of FGR, but a baseline characteristic in GRN. Since GRN is the postpartum equivalent of FGR, the alignment of these sets enables future studies to compare data deriving from the same pathological condition, but investigated at different moments (antenatal and postnatal).

### Interpretation

The COS and MRS are the minimum of what should be reported in future studies focused on GRN and can be used as a framework for the outcome and baseline characteristic selection. Future work will focus on how these outcomes should be defined and reported. Until further data are available, we encourage researchers to clearly report the measures that they have used. In our opinion, outcomes included in a COS are not mandatory to be measured if for that study specifically it is unfeasible; however, if measured or assessed for clinical reasons, the outcome should be reported. If not assessed, it should be indicated why the outcome is not applicable for transparency and to reduce the risk of reporting bias.

Of note, it is essential that trial populations of fetal and neonatal growth are critically reviewed for their definition of the diagnosis, along with the use of the developed COS and MRS. In the absence of a golden standard to diagnose FGR and GRN, inconsistency in definitions is a problem in interpreting and comparing data. To overcome heterogeneous defining and reporting of the diagnosis, international consensus definitions were developed. Current sets apply to growth-restricted newborns defined according to these international consensus definitions.^1,20^

## Conclusion

In the COSNEON procedure both a COS and an MRS were developed that, as a minimum, should be collected in future studies with a focus on GRN. This will help standardize reporting on this topic and facilitate the comparison of data across studies to guide clinical practice.

## Supplementary information

Supplementary tables
