# Supplementary material for: A Core Outcome Set and minimum reporting set for intervention studies in growth restriction in the NEwbOrN: the COSNEON study
Source: Pediatr Res. 2020 Sep 14;89(6):1380–5. doi: 10.1038/s41390-020-01119-5 (PMC8163598; doi:10.1038/s41390-020-01119-5)
Supplement: Supplementary file 1 — Supplementary tables [file 41390_2020_1119_MOESM1_ESM.docx]

**Supplemental table S1** Outcomes and baseline characteristics removed or combined at the consensus meeting

| *Item* | *Consensus meeting* |
| --- | --- |
| **Core Outcome Set** |  |
| Hypoxic Ischaemic Encephalopathy | Removed from COS and included in MRS |
|  |  |
| **Minimum Reporting Set** |  |
| Gestational hypertension | Aggregated into ‘hypertensive disorders during pregnancy’ |
| Preeclampsia | Aggregated into ‘hypertensive disorders during pregnancy’ |
| HELLP | Aggregated into ‘hypertensive disorders during pregnancy’ |
|  |  |
| Infection | Specified into ‘congenital infection likely to affect fetal growth’ |

**Supplemental table S2** Outcomes excluded from the final COS

| *Domain* | *Outcome* |
| --- | --- |
| **Gastrointestinal complications** | Cholestasis |
|  | Gastrointestinal perforation |
|  | Ileus |
| **Respiratory / lung complications** | Apnoea |
|  | Episodes of desaturation <80% for at least 2 seconds |
|  | Need for ventilatory support |
|  | Respiratory distress syndrome |
| **Neurological complications** | Meningitis |
|  | Seizure |
| **Heart / vascular complications** | Bradycardia |
|  | Patent ductus arteriosus |
|  | Pulmonary hypertension |
| **Infection and other** | Infection |
|  | Metabolic abnormalities |
|  | Retinopathy of prematurity |
| **Feeding outcomes** | Amount of nutrition intake (ml/kg/day) |
|  | Duration of IV fluids |
|  | Duration of nasogastric tube |
|  | Duration of parenteral nutrition |
|  | Feed fortification |
|  | Formula milk |
|  | Full enteral feeding sustained (days) |
|  | Mixed breast feeding and formula milk |
|  | Need for IV fluids |
|  | Need for nasogastric tube |
|  | Need for parenteral nutrition |
|  | Time between delivery and the first enteral feeding |
|  | Time to achieve full enteral feeding |
| **Feeding intolerance** | Abdominal distension |
|  | Diarrhoea |
|  | Duration of episode of feeding intolerance (days) |
|  | Episodes off feeding intolerance |
|  | Frequency of episodes of feeding intolerance |
|  | Gastric residuals |
|  | Gastroesophageal reflux |
|  | Macroscopic blood in stools |
|  | The feed volume at which intolerance becomes apparent |
|  | Vomiting |
|  | Withheld feedings |
| **Stool** | Initial stool frequency per day |
|  | Initial stool frequency per week |
| **Hospital and mortality** | Normal abdominal ultrasound |
|  | Normal cerebral ultrasound |
|  | Receiving blood transfusion |
| **Need for medication use of the newborn** | Need for antibiotics |
|  | Need for caffeine or aminophylline |
|  | Need for inotropic support |
|  | Need for steroids |
| **Growth / weight** | Initial weight loss greater than 10% |
|  | Postnatal growth failure |
| **Blood laboratory results** | Anaemia |
|  | Hyperbilirubinemia |
|  | Hypoglycaemia |
|  | Infection parameters (CRP/Leucocytes) |
| **Long term follow up** | ADHD |
|  | Bayley III score |
|  | Need for special education |

**Supplemental table S3** Excluded baseline characteristics from the MRS

| *Domain* | *Baseline characteristic* |
| --- | --- |
| **Baseline characteristics** | Maternal ethnicity |
|  | Paternal ethnicity |
|  | Paternal highest level of education |
| **Obstetric information** | Gravida |
|  | History of earlier pregnancy with FGR or GRN |
|  | Intends to breastfeed |
|  | Parity |
|  | Previously breastfed |
|  | Use of assisted reproductive technology |
| **Complications antenatal** | Chorioamnionitis |
|  | Placental abruption |
|  | Premature rupture of membranes |
|  | Pulmonary embolism |
| **General information postpartum** | APGAR score |
|  | Cardiac resuscitation just after birth |
|  | Caesarean section |
|  | Congenital anomalies not likely to affect fetal growth |
|  | Ethnicity |
|  | Induction of labour |
|  | Insufflation just after birth |
|  | Knee-heel lengths at birth (centimetres) |
|  | pH umbilical artery |
|  | pH umbilical vein |
|  | Place of birth (i.e. Hospital, home) |
|  | Reason for caesarean section |
|  | Reason for induction of labour |
|  | Stimulation just after birth |
| **Scores** | CRIB |
|  | SNAPPE II |
|  | Thompson score |

APGAR Appearance, Pulse, Grimace, Activity, and Respiration

CRIB Clinical risk index for babies

FGR Fetal growth restriction

GRN Growth restriction in the newborn

SNAPPE Score for neonatal acute physiology-perinatal extension
